# 

**Published:** 1954

**Authors:** 


MEDICAL JOURNAL OF THE SOUTH-WEST

*(Incorporating the Bristol Medico-Chirurgical Journal)*

VOL. 70 (ii) 1954 NO. 254

### Contents

*PUBLISHED QUARTERLY*

*ANNUAL SUBSCRIPTION 16/- POST FREE*

PUBLISHED UNDER THE AUSPICES OF THE

BRISTOL MEDICO-CHIRURGICAL SOCIETY

### Editorial Committee

MR. H. KEITH LUCAS, 60 St. John's Road, Bristol 8. *Editor.*

DR. J. E. CATES, Dept. of Medicine, Bristol Royal Infirmary } *Assistant Edit*[illegible]

DR. A. H. GALE, The University, Bristol 8

DR. J. MACRAE, Ham Green Hospital. *Treasurer.*

DR. A. M. MACLACHLAN, 167 Redland Road, Bristol 6.

DR. F. J. Y. WOOD, Dept. of Medicine, Bristol Royal Infirmary.

### *Local Correspondents*

*Bath* . . Mr. A. D. BATEMAN, 3 The Circus, Bath.

*Exeter* . . Dr. L. N. JACKSON, Mount Jocelyn, Crediton, Devon.

*Gloucester* . Dr. R. F. JARRETT, 9 College Green, Gloucester.

*Plymouth* . Dr. C. R. CROFT, Lexden, Hartley Av., Plymouth.

*Taunton* . Dr. M. MCALLEY, 1 The Crescent, Taunton.

*Torquay* . Dr. A. ROBINSON THOMAS, 20 Devon Square, Newton Abbot, Dev[illegible]

*Truro* . . Dr. F. D. M. HOCKING, St. Andrews, Perranporth, Cornwall.

*Yeovil* . . Mr. J. A. VERE NICOLL, Knapp House, Yeovil, Somerset.

### NOTICE TO CONTRIBUTORS

Original articles are invited, provided they have not been published elsewhere. N[illegible] of forthcoming events, meetings held, degrees conferred, staff appointments, and o[illegible] local news are welcomed. The editorial committee reserves the right to make liter[illegible] corrections.

Illustrations should always be supplied ready for reproduction. All re-touchings [illegible] other operations required will be charged to the author. Line drawings should be dra[illegible] in Indian ink. We regret that we must ask authors to pay for making blocks, if thi[illegible] necessary.

J. W. ARROWSMITH LTD., SMALL STREET,

BRISTOL.

### *Appointments*

### South Western Regional Hospital Board

### *CONSULTANTS, SPECIALISTS AND REGISTRARS APPOINTED DURING THE PERIOD OCTOBER TO DECEMBER*, 1953

### BRISTOL

| *Name* | *Appointment* | *Formerly* |
|---|---|---|
| CHAMPION, ELIZABETH P. (Mrs. Bassett), M.B., B.S., (SYDNEY), D.A. (LONDON). | Anaesthetic Registrar, Southmead Hospital. | Locum Consultant Anaesthetist, Harts Hospital, Woodford. |
| ILES, KATHLEEN C., M.B., CH.B.(BRISTOL). | Registrar in Diseases of the Chest, Bristol. | J.H.M.O., Royal National Hospital for Diseases of the Chest, Ventnor, I.O.W. |
| RICHARD, D. R., B.M., B.CH. | Surgical Registrar, Weston-super-Mare General Hospital. | Locum S.H.O. in General Surgery, St. Alfeges Hospital, Greenwich. |
| ARTHURS, G., L.R.C.P., M.R.C.S., M.B., B.S., F.R.C.S. | Senior Registrar in Neurosurgery, Frenchay Hospital. | Senior Registrar in Neurosurgery, Morriston Hospital, Swansea. |
| DUTTON, J. E. M., M.B., B.S., F.R.C.S. | Registrar in Neurosurgery, Frenchay Hospital. | S.H.O., Neurosurgical Unit, Frenchay Hospital. |
| SEAGER, C. P., B.SC., M.B., B.CH. | Psychiatric Registrar, Bristol Mental Hospitals. | S.H.O., Bristol Mental Hospitals. |
| JOYCE, M. H. B., M.B., CH.B. | Psychiatric Registrar, Bristol Mental Hospitals. | S.H.O., Bristol Mental Hospitals. |
| HEATON-WARD, W. A., M.B., CH.B. (BRISTOL), D.P.M. (BRISTOL). | Medical Superintendent, Stoke Park Colony Group. | Deputy Medical Superintendent, Hortham–Brentry Hospital Group. |

### NORTH GLOUCESTERSHIRE

| *Name* | *Appointment* | *Formerly* |
|---|---|---|
| MARTIN, L. V., M.B., B.S., D.A. | Consultant Anaesthetist, North Glos. Clinical Area. | Senior Registrar in Anaesthetics, Welsh National School of Medicine, Cardiff. |
| FOREMAN, J. M., M.B., CH.B. (NEW ZEALAND), F.R.C.S.E. | Surgical Registrar, Glos. Royal Hospital. | Surgical Registrar, Newcastle General Hospital. |
| SANDERS, E. J., M.B., B.CH. (CAPE TOWN). | Surgical Registrar, Cheltenham General Hospital. | H.S., General Surgical Unit, Oldcurch Hospital, Romford. |
| VINCENT, C. T., M.R.C.S., L.R.C.P., M.B., B.S. | Assistant Clinical Pathologist (S.H.M.O.), North Glos. Clinical Area. | Senior Pathological Registrar, Lambeth Group Laboratory. |

### BATH

| *Name* | *Appointment* | *Formerly* |
|---|---|---|
| WELLS, C. E. C., M.B., B.S., M.R.C.P. | Senior Medical Registrar, Bath Clinical Area. | Medical Registrar, Southmead Hospital. |
| MUSCAT, S., B.SC. (MALTA), M.D. (MALTA), D. (OBST.), R.C.O.G. | Registrar in Obstetrics and Gynaecology Bath Group of Hospitals. | S.H.O., Obstetrics and Gynaecology Dept., Farnborough Hospital. |
| MORGAN, JEAN E., M.B., CH.B. | Pathological Registrar, Bath Group of Hospitals. | S.H.O., Dept. of Pathology, Frenchay Hospital. |

### SOUTH SOMERSET

| *Name* | *Appointment* | *Formerly* |
|---|---|---|
| PRETTEJOHN, E. J. T., M.B., B.CHIR., M.R.C.P. | Consultant Dermatologist, South Somerset Clinical Area (three sessions). | Senior Registrar in Dermatology, United Bristol Hospitals. |
| HARKNESS, J., B.SC., M.B., CH.B., M.D. | Area Pathologist, South Somerset Clinical Area. | Consultant Pathologist, Central Laboratory, Portsmouth. |
| CUTHILL, J. M., M.B., CH.B., D.P.M. | Senior Registrar in Psychiatry, Tone Vale Hospital. | Deputy Specialist i/c Neuro-psychiatric Centre, Princess Mary's R.A.F. Hospital. |

### EXETER

| *Name* | *Appointment* | *Formerly* |
|---|---|---|
| FREEMAN, A. G., M.R.C.S., L.R.C.P., M.A., M.B., B.CHIR., M.D., M.R.C.P. | Senior Medical Registrar, Exeter Clinical Area. | Assistant Chest Physician (Locum), Cardiff and District. |
| CANDLER, T. O., M.B., B.CH., M.R.C.S., L.R.C.P., F.R.C.S. | Clinical Assistant in Orthopaedic and Traumatic Surgery, Bideford and District Hospital (one session). | In general practice, Bideford. |

### WEST CORNWALL

| *Name* | *Appointment* | *Formerly* |
|---|---|---|
| PENGELLY, C. D. R., M.B., CH.B., M.R.C.P.E. | Senior Medical Registrar, West Cornwall Clinical Area. | Medical Registrar, United Bristol Hospitals. |
| LAKEMAN, MARGARET A., M.R.C.S., L.R.C.P., D. (OBST.), R.C.O.G., M.B., B.S. | Registrar in Obstetrics and Gynaecology, Camborne–Redruth Miners' and General Hospital, Redruth. | H.S., Whittington Hospital, London. |

### *Notes and News*

### POSTGRADUATE COURSES FOR GENERAL PRACTITIONERS

The following courses are being arranged:

1. *May 14th to 17th:* Week-end course at Exeter.

2. *July 11th to 17th:* Intensive course at Bristol in Medicine, Surgery and Paediatrics.

3. *April–June:* Sunday morning lecture demonstrations in Bristol.

Detailed programmes may be obtained later from: Director of Postgraduate Medical Studies. Telephone: Bristol 24161. Extension 7.

### MEDICAL EXHIBITIONS

The Organizers of the London Medical Exhibition have recently announced plans for their 1954 provincial Exhibition, to be held at Victoria Rooms, Bristol University, from May 17th to 21st, inclusive.

Information relating to this and the London Exhibition should be obtained direct from: The Organizers, London Medical Exhibition, 194–200, Bishopsgate, London, E.C.2.

### WESSEX RAHERE CLUB

The Spring Dinner of the above Club will take place at the Royal Clarence Hotel, Exeter, on Saturday, April 24th.

Mr. H. Jackson Burrows, F.R.C.S. has kindly accepted the invitation to be present as Guest of Honour.

Membership of the Club is open to all Barts men practising in the West Country. Further details will be circulated to Members and to any other Barts men who are interested and who will get in touch with the Hon. Secretary, Mr. A. Daunt Bateman, of 11 The Circus, Bath.

